# A wavelet-based technique to predict treatment outcome for Major Depressive Disorder

**DOI:** 10.1371/journal.pone.0171409

**Published:** 2017-02-02

**Authors:** Wajid Mumtaz, Likun Xia, Mohd Azhar Mohd Yasin, Syed Saad Azhar Ali, Aamir Saeed Malik

**Affiliations:** 1 Centre for Intelligent Signal and Imaging Research (CISIR),Universiti Teknologi PETRONAS, Bandar Seri Iskandar, Perak, Malaysia; 2 Beijing Institute of Technology, Beijing, China; 3 Department of Psychiatry,Universiti Sains Malaysia, Jalan Hospital Universiti Sains Malaysia, Kubang Kerian, Kota Bharu, Kelantan, Malaysia; National University of Defense Technology College of Mechatronic Engineering and Automation, CHINA

## Abstract

Treatment management for Major Depressive Disorder (MDD) has been challenging. However, electroencephalogram (EEG)-based predictions of antidepressant’s treatment outcome may help during antidepressant’s selection and ultimately improve the quality of life for MDD patients. In this study, a machine learning (ML) method involving pretreatment EEG data was proposed to perform such predictions for Selective Serotonin Reuptake Inhibitor (SSRIs). For this purpose, the acquisition of experimental data involved 34 MDD patients and 30 healthy controls. Consequently, a feature matrix was constructed involving time-frequency decomposition of EEG data based on wavelet transform (WT) analysis, termed as EEG data matrix. However, the resultant EEG data matrix had high dimensionality. Therefore, dimension reduction was performed based on a rank-based feature selection method according to a criterion, i.e., receiver operating characteristic (ROC). As a result, the most significant features were identified and further be utilized during the training and testing of a classification model, i.e., the logistic regression (LR) classifier. Finally, the LR model was validated with 100 iterations of 10-fold cross-validation (10-CV). The classification results were compared with short-time Fourier transform (STFT) analysis, and empirical mode decompositions (EMD). The wavelet features extracted from frontal and temporal EEG data were found statistically significant. In comparison with other time-frequency approaches such as the STFT and EMD, the WT analysis has shown highest classification accuracy, i.e., *accuracy = 87*.*5%*, *sensitivity = 95%*, *and specificity = 80%*. In conclusion, significant wavelet coefficients extracted from frontal and temporal pre-treatment EEG data involving delta and theta frequency bands may predict antidepressant’s treatment outcome for the MDD patients.

## Introduction

Major depressive disorder (MDD), also termed as depression, is a common mental illness that is life threatening, progressive, recurrent, and may cause functional disabilities. In USA, a high prevalence among elderly patients (age: 50+ years) has been observed ranging from 13.2% to 16.5% [1]. In addition, MDD has been associated with low treatment efficacy as investigated in a study known as sequenced treatment alternative to relieve depression (STAR*D) [2, 3]. The study concluded with a response rate, i.e., 47% which was even less than half the total study participants. Selective Serotonin Reuptake Inhibitors (SSRIs), including more than 2 dozen antidepressants, are considered as first-line treatment selection for MDD [4]. However, due to heterogeneity of the condition, an appropriate selection of antidepressants, early during patient care, remains an elusive goal for MDD. In case of treatment failure, an adequate period of 2 to 4 weeks is wasted and a reselection is also based on minimal scientific evidence.

Successfully predicted antidepressant’s treatment outcomes early during patient’s care could improve the low treatment efficacy associated with antidepressants. In this context, the electroencephalogram (EEG)-based research studies have shown promising results and can be reviewed elsewhere [5–7]. EEG offers high temporal resolution and low cost which makes it suitable for applications such as monitoring epileptic patients [8, 9], quantification of sleep stages [10], and monitoring anesthesia dosage [11]. In the literature, various studies have proposed EEG features to predict antidepressant’s treatment outcome, for example, spectral power estimation for EEG alpha and theta frequency bands [12, 13], alpha asymmetry [14, 15] and theta power [16, 17]. In addition, combinations of EEG features including signal powers at alpha and theta frequency bands are proposed, e.g., the antidepressant treatment response (ATR) index [18] and the EEG theta cordance [19]. The ATR index had achieved 70% accuracy for classifying treatment responders (R) and non-responders (NR) [18]. In addition, similar findings are endorsed in different studies [20, 21]. Furthermore, studies based on EEG theta cordance have reported a consistent observation, i.e., a decreased prefrontal theta cordance associated with treatment response [12, 19, 22, 23]. The research results implicate that both the ATR index and the EEG theta cordance are promising methods. However, their clinical utility has been largely understudied because they have demonstrated low values of specificity.

Referenced EEG (rEEG) is a technique involving a database of MDD patient’s EEG patterns and medical treatment histories [24, 25]. The EEG patterns are used to guide the selection of suitable antidepressants for a new MDD patient visiting the facility. The rEEG-based research studies have shown improved treatment results than the STAR*D studies [24, 26]. However, rEEG is less explored clinically and may need more research efforts. Furthermore, the EEG-based brain source estimation technique such as the LORETA (LOw Resolution brain Electromagnetic Tomography Analysis) is used to localize neuronal sources deep inside the brain and explored associations between the activated (based on current density) brain areas and antidepressant’s treatment outcome. For example, the activations found in rostral anterior cingulate cortex (rACC) are associated with antidepressant treatment responders [27–30]. Recently, machine learning (ML) techniques have shown 85% and 87.9% accuracies as treatment outcome prediction for schizophrenic and MDD patients [31–33]. The ML techniques have utilized various EEG features as input data such as coherence, mutual information between any 2 EEG sensors, power spectral density (PSD), and PSD ratios [31].

In summary, research studies based on utilizing EEG features to predict antidepressant’s treatment outcome for MDD have shown their promises, termed as EEG biomarkers [34]. However, the EEG biomarkers for MDD could not prove their clinical utility due to certain limitations such as low specificities, small sample sizes, less generalizability and large scale replications. Hence, more solid and systematic research efforts are needed that could result into high values of sensitivities and specificities. This could be achieved with carefully selected study participants such as having balanced gender distribution, large enough samples that reflect the whole population and utilizing robust EEG features as input data to develop robust ML methods.

The time-frequency decomposition of EEG data involves multiple techniques, such as, the wavelet transform (WT) analysis [35, 36], empirical mode decomposition (EMD) [37], and the short-time Fourier transform (STFT) analysis [38]. However, the time-frequency decomposition of EEG data has not been investigated to generate predictions for antidepressant’s treatment outcome for MDD. For example, the WT analysis has been utilized into various medical applications [39, 40] including diagnosing Epilepsy and Alzheimer [9, 41]. In a study, both STFT and WT analysis are used for electrocardiogram (ECG) analysis to extract information/features [42]. However, the STFT is unable to provide a detailed analysis. In contrary, the WT analysis successfully extracts the desired information due to well-capturing the EEG signal nonlinearities than fixed window functions employed by STFT analysis. Hence, the WT analysis performs better than STFT analysis during feature extraction. In addition, the authors concluded that the WT analysis provides more robust features than STFT in order to characterize ECG signals and to help physicians obtaining the qualitative and quantitative measurements.

The WT analysis utilizes predefined window functions at customized frequencies and time scales [43]. However, the selection of a window function is subjective and depends on the type of analysis and underlying EEG data. For example, in a study, the wavelet window function ‘db4’ is found appropriate for analyzing EEG data [44]. Moreover, the WT analysis has the ability to compute or manipulate the data into compressed parameters, termed as features that may help reduce irrelevant information and characterize the behavior of EEG. The WT analysis is implemented based on filter banks approach that include low and high filter branches.

In EMD, the EEG data are decomposed into intrinsic mode functions (IMFs) without a preselected window function. Instead the window functions are constructed based on the maximum and minimum values of the underlying EEG data. The original EEG signal decomposed into various IMFs represent different time-scales and frequency bandwidths [45]. For example, the first IMFs correspond to high frequency components. On the other hand, the last IMFs represent low frequencies, termed as the residues. In EMD, the frequency is derived by differentiation rather than by convolution, as for the WT analysis; this allows to overcome the limitations of uncertainty principle, and hence solves intrinsic limitation of WT analysis [43]. On the other hand, the EMD lacks theoretical foundation because of its empirical nature. However, both WT analysis and EMD might be able to cope with possible non-linearity of the EEG signals.

In this study, a ML method is proposed that involves feature extraction, selection, classification, and 10-fold cross validation (10-CV). The EEG data are decomposed with WT analysis in order to classify the MDD patients into responders (R) and non-responders (NR). In addition, the same EEG features are used to classify the MDD patients and healthy controls. Furthermore, replication of previous work is performed by identifying best feature from EEG and event-related potential (ERP) data found in the related literature, e.g., alpha and theta powers, alpha asymmetry, ATR index, EEG theta cordance, coherence and the ERP components such as P300 amplitudes and latencies.

## Materials and methods

### Study participants

In this research, a sample of 34 MDD outpatients (17 males and 17 females, mean age = 40.3 ±12.9) was recruited according to the experiment design approved by the human ethics committee of the Hospital Universiti Sains Malaysia (HUSM), Kelantan, Malaysia. The study participants were able to sign the consent forms of participation and were briefed about the experiment design. The MDD patients met the internationally recognized diagnostic criteria for depression, named as Diagnostic and Statistical Manual-IV (DSM-IV) [46]. Table 1 provides statistics regarding patient’s age, gender and pre- and post-treatment disease severity scores, the sample size calculation, and the study’s inclusion and exclusion criteria. In addition, Table 2 illustrates the diagnosis information of the MDD patients. The diagnosis information reflected the patient’s conditions at the time of recruitment. In order to avoid medication effects, the MDD patients had gone through a washout time period of two weeks before commencing the 1st EEG recording. The MDD patients started taking antidepressants under the general category of SSRIs with psychiatrist’s consultation.

**Table 1:** Summary of MDD patient’s clinical characteristics.

Information	R	NR	Total
**Age [years]**	40.7 (±13.0)	41.1 (±12.5)	40.3 (±12.9)
**Gender (female/male)**	8/8	9/9	17/17
**Pretreatment BDI [47]**	18.4 (±7. 4)	22.8 (±12.5)	20.6 (±8.6)
**Pretreatment HADS [48]**	11 (±1.5)	10.4 (±3.2)	10.7 (±2.4)
**Post-treatment BDI-II**	9.1 (±6.3)	22.1 (±3.3)	15.6 (±4.5)
**Post-treatment HADS**	5.9 (±4.7)	10.1 (±5.1)	7.5 (±5.0)
^**1**^**SSRI treatment**	E:9,F:2,S:4,Fl:1	E:5,F:7,S:4,Fl:2	E:14,F:9,S:8,Fl:3
**Sample Size Calculation**	A group of thirty four (34) MDD patients will be recruited based on formula given below [49, 50]: $n=\frac{P(1-P).{\left(Z_{1-\alpha /2}\right)}^{2}}{e^{2}}$ where *P* is the expected proportion (e.g., expected diagnostic sensitivity), *e* is the error limit which is one half the desired width of the confidence interval, and *Z*_1−*α*/2_ is the standard normal Z value corresponding to a cumulative probability of 1−*α*/2. The investigator must specify the best guess for the proportion that is expected to be found after performing the study [2]. For the research project study following are the parameter values.—significance α = 0.05 (alpha)—power of test = 80%, β = 0.2 (Beta)—expected diagnostic accuracy P = 90% [31]—expected error e = 10% $P=0.90,\alpha =0.05,e=0.10,Z_{1-\alpha /2}=1.96\;n=\frac{(0.90)(0.10){\left(1.96\right)}^{2}}{{\left(0.10\right)}^{2}}≅34$		
**Inclusion Criteria**	1)Able to provide written informed consent		
	2)Patients with Age (18–65 years)		
	3)Patients Diagnosed MDD (DSM-IV)		
	3a)Newly Diagnosed (New Cases)		
	3b)Newly Started (Old Cases)		
	4)Re-Started On Antidepressant (Two Week Washout)		
	4a)Switched To New Antidepressant		
**Exclusion Criteria**	1- Patients having psychotic, cognitive disorder		
	2- Patients with any other drug abuse		
	3- Pregnant patients		
	4- Patients with epilepsy		

^1^ SSRI medication administered: E: Escitalopram 10–20 mg per day, F: Fluvoxamine 100–300 mg per day, S: Sertraline 50–200 mg per day, Fl: Fluxetine 20–60 mg per day.

**Table 2:** Diagnosis information of MDD patients with comorbidities [51].

**Percentage of Patients With Major Depression Who Also Suffer From a Current Anxiety Disorder**			
	**Percentage [51]**	**Responders**	**Non-responders**
**Anxiety disorder**	51%	11	12
**Social phobia**	20%	2	3
**Generalized anxiety disorder (GAD)**	15.4%	1	1
**Post-traumatic stress disorder (PTSD)**	15.2%	2	0
**Panic/Agoraphobia**	12.6%	1	1
**Rates of Depression in Patients With Medical Illness**			
	**Percentage [51]**	**Responders**	**Non-responders**
**Cancer**	20.5%	0	0
**Coronary artery disease or Myocardial infarction**	15.4%	0	0
**Stroke**	20.4%	0	0
**Parkinson disease**	30.4%	0	0

Moreover, a second group of 30 age-matched healthy controls (21 males and 9 females, mean age = 38.3±15.6) were recruited as a control group. The healthy participants were examined for psychiatric conditions and were found healthy.

### Definition of response

During each visit to the clinic, the MDD patients were assessed by experienced clinicians based on two questionnaires, i.e., Beck Depression Inventory-II (BDI-II) [47] and Hospital Anxiety and Depression Scale (HADS) [48]. After 4^th^ week, the MDD patients were labeled as ‘R’ and ‘NR’ based on the scores observed from BDI-II and HADS, and the scores were considered as gold standard during EEG analysis. According to the treatment algorithm for MDD published by the Malaysian Psychiatric Association (MPA), at-least four weeks of treatment, termed as adequate period, is required before making any assessment of the treatment [52]. However, in this study, the MDD patients were followed for six weeks after starting medication. Table 1 shows the observed changes monitored with BDI-II and HADS.

In this study, there were multiple reasons to select BDI-II and HADS instead of the Hamilton rating scale for depression (HAM-D) and Montgomery-Asberg Depression Scale (MADRS). Firstly, the HADS and BDI-II have been considered as the standard clinical tools to assess the severity of depression. Secondly, properly validated Malay version of BDI-II [47] and HADS [48] were available and could easily be understood by the local population of MDD patients.

In the literature, the response to treatment with SSRIs has been consistently reported ranging from 50% to 60% [53–57]. In this study, the response to treatment was defined as a 50% improvement in clinical symptoms assessed with the BDI-II scores, i.e., a 50% improvement in pre- vs. post-treatment BDI-II scores. According to the BDI-II, a study participant was considered as normal for an accumulated score ranging from 0 and 10; as mildly depressed for scores range from 11 to 20; as moderately depressed for scores ranging from 21 to 30; as severely depressed for 31 to 40; very severely depressed for 41 to 63. In addition, according to HADS, the cumulative scores greater than seven (>7) is considered as abnormal.

### EEG data acquisition

**Fig 1:**
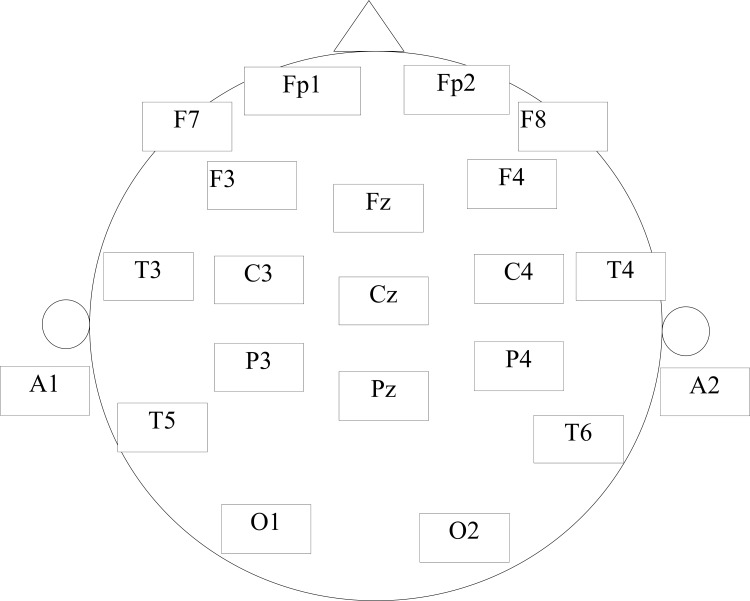
The EEG cap from Brain Master Discovery, employed sensors placed according to the internationally recognized 10–20 electrode placement standard.

As shown in Fig 1, EEG cap with nineteen (19) electro-gel sensors was used to acquire EEG data. The electro-gel sensors required fewer adjustments than the hydro-sensors; hence, facilitating longer recordings and enhanced patient care. In this study, the on-scalp placements of the EEG sensors followed the international 10–20 system [58]. According to the 10–20 system, the sensors can be categorized into different regions, e.g., the frontal included 7 electrodes: Fp1, F3, F7, Fz, Fp2, F4, and F8. In addition, the central included C3, C4 and Cz; the parietal lobe included P3, Pz and P4; the occipital involved O1, O2 and the electrodes T3, T4, T5, T6 cover left and right temporal region.

In this study, the EEG data were recorded with Linked ear (LE) reference and were re-referenced to the Infinity reference (IR) [59]. The EEG data recorded with LE reference can be re-referenced as Average reference (AR) and IR. In the literature, the AR and IR were recommended as equally efficient [60, 61]. However, none of the methods were considered as gold standard [60].

An amplifier named Brain Master Discovery (Make: Brain Master, Model: Discovery 24e, Manufacturer: Brainmaster Technologies Inc.) was used to amplify the weak EEG signals from the sensors. Furthermore, the EEG data were digitized with 256 samples per second, band pass filtered from 0.1 to 70 Hz with an additional 50 Hz notch filter to suppress power line noise.

The EEG data were recorded at pretreatment (before start of medication) and after each week until the completion of the study duration (6 weeks). In this study, the pre-treatment EEG data were used to perform EEG-diagnosis and EEG-based prediction of treatment outcome and were considered as the main contribution of the paper. However, the EEG data recorded at week 1 (after the medication started) and the ERP data recorded at week 0 (pretreatment) were used to replicate the prior art. The details on the EEG and ERP data are provided below.

The EEG data were recorded during eyes closed (EC) (5 minutes) and eyes open (EO) (5 minutes) conditions while the study participants (MDD patients and healthy controls) were instructed to sit in a semi-recumbent position with minimal eye blinks and head movements.

**Fig 2:**
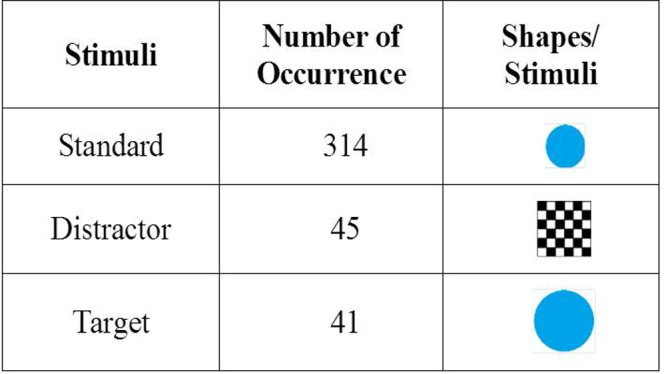
Shapes used during the 3-stimulus visual Oddball Task.

The ERP data were recorded for ten (10) minutes involving a 3-stimulus visual oddball task [62]. The study participants were exposed to a computer screen displaying a random sequence of shapes (as shown in Fig 2). A total of three (3) shapes were used named as the Target (a blue circle with 5.0 cm size), the Standard (a blue circle with 4.5 cm size), and the Distractor (a checker board with 18.0 cm size). The shapes were displayed on the computer screen randomly and one-by-one for 400 times such as the Standard, the Distractor, and the Target shapes were appeared for 314, 45, and 41 times, respectively. The display time for a stimulus was 1.5 seconds involving display of the shape (0.5 second) and display of a fixation window (1 second). The participants were instructed to press the SPACE key on a keyboard only when the Target shape appeared. On the other hand, they were instructed to remain idle during the occurrences of the Standard and the Distractor shapes.

Finally, both the EEG and ERP data were saved on a computer disk for noise reduction (EEG pre-processing) and analysis (ML process).

### EEG pre-processing

Artifact-free EEG data were desirable to avoid erroneous subsequent analysis and to make sure that the data truly represent the underlying neuronal activity. Therefore, in this study, the EEG preprocessing involved correction of artifacts due to eye movements (horizontal and vertical), blinks, muscular, and heart activities. Moreover, the artifact corrections were performed with standard tools including adaptive and surrogate filtering techniques, implemented in brain electrical source analysis (BESA) software [63]. A similar procedure of artifact correction was adopted for all study participants including the MDD patients and the healthy controls.

In BESA, cleaning EEG data (artifact types: eyes blinks, muscle activity, line-noise, heart activity, etc.) was based on a semi-automatic procedure, the technique has the name multiple source eye correction (MSEC) [64]. According to the technique, the raw EEG data were used to first estimate noise topographies. An appropriately selected head model (selected in BESA) and the noise topographies were used further to correct the artifacts. According to the procedure, an investigator needed to select the type of artifact (artifact types: eyes blinks, muscle activity, line-noise, heart activity, etc.) to be corrected. The selection allowed the software to mark the artifacts in the whole EEG recording. The marking of artifacts facilitated further to estimate the noise topographies generated by BESA. The procedure was repeated for all kinds of artifact types including the artifacts due to the eye-blinks, eye movements, muscular, and heart activity. Hence, the artifacts found in the raw EEG were corrected.

### Overview of ML process

**Fig 3:**
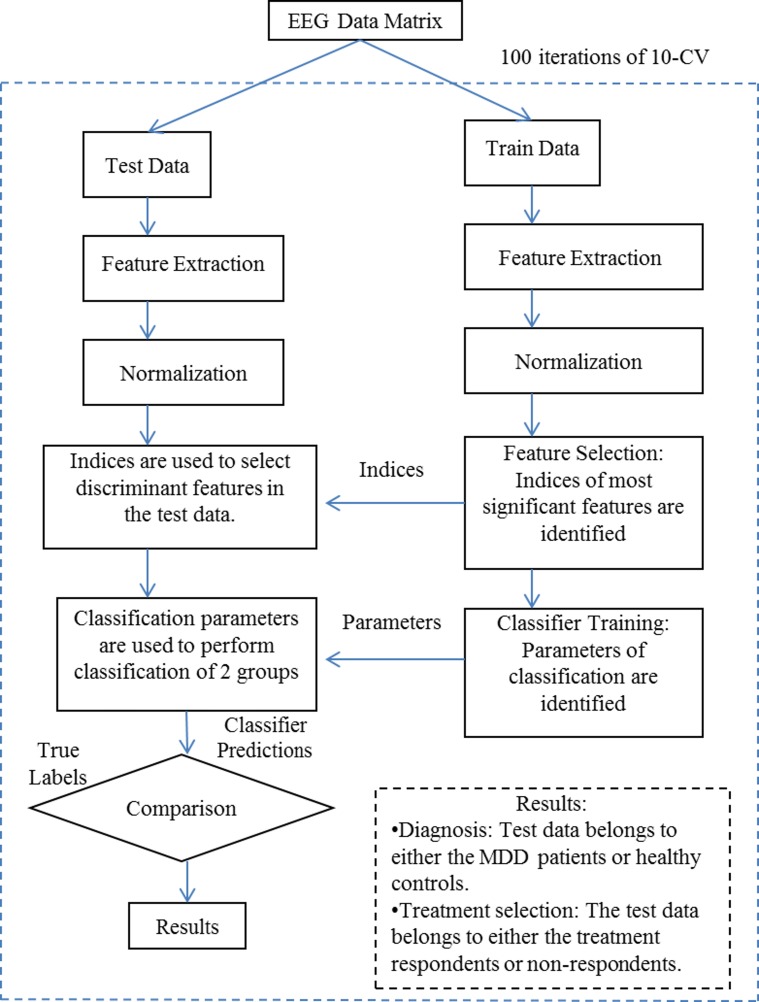
Overview of the ML scheme for EEG data analysis.

Fig 3 shows the proposed ML method that involved pretreatment EEG-based features as input data to classify the study participants into either *‘R’* or *‘NR’*. The input data involved WT analysis including two minutes of each of the clean EC and EO data. Two (2) minutes of resting-state EEG data has been considered as sufficient to extract the useful information. In this study, different lengths (1, 2, and 3 minutes) of the EEG segments were considered during computation of the features, e.g., the PSD. We have observed slightly better results for 2 minutes of EEG data than 1 minute of EEG data. However, there are no considerable changes observed in the performances between 2 minutes of EEG data and 3 minutes of EEG data. Hence, in this study, the results were reported for EEG data of 2 minutes length.

The feature extraction resulted into a large number (*N*_*c*_) of candidate features and were arranged column-wise in a matrix, termed as EEG data matrix. Each column of the data matrix represented a feature/variable and denoted as *x*_*i*_, where *i = 1… N*_*c*_. In the matrix, rows represented MDD patient’s EC and EO data, termed as instances/examples. The feature space denoted by *L = [(x*_*i*_,*y*_*i*_*)*, *i = 1 … N*_*c*_*]* included both the EEG data matrix and the corresponding output class labels or targets, *y = [R*, *NR]*. To determine the effects of EEG data lengths, EEG segments of one and two minutes were used to compute the classification results.

As shown in Fig 3, the EEG data matrix was divided into train and test sets according to the 10-CV. The iterations of 10-CV ensured independence of the train and test sets and the feature selection and building classification model were performed based on the training sets only. On the other hand, the selection of features in the test set involved the feature indices already identified from the train set. Hence, the training process including feature selection and building classification model was performed independent of the test data. Similarly, the feature normalization (z-transformation) was performed separately for the train and test sets [65].

In this study, the proposed ML process involved feature extraction, selection, classification, and validation. The feature extraction included multi-resolution decomposition of EEG data with WT analysis. Moreover, two similar techniques, i.e., EMD, and STFT were also employed for comparison purposes. Hence, the EEG decomposition resulted into three different EEG data matrices. To reduce the dimensionality of the input EEG data matrices, the feature selection was performed with two techniques: 1) rank-based feature selection according to their relevance with the class labels (R Vs. NR and MDD patients Vs. healthy controls) based on a criterion known as receiver operating characteristics, i.e., roc [66], and 2) minimum redundancy and maximum relevance (mRMR) method [67]. In the proposed ML scheme, the rank-based feature selection method was used to select most significant features from the EEG data matrix. To validate the rank-based feature selection method, it was compared with the mRMR method. Finally, the discriminant EEG features were identified and used as input data to train and test the LR classifier involving 100 iterations of 10-CV.

#### Feature extraction: WT analysis

**Fig 4:**
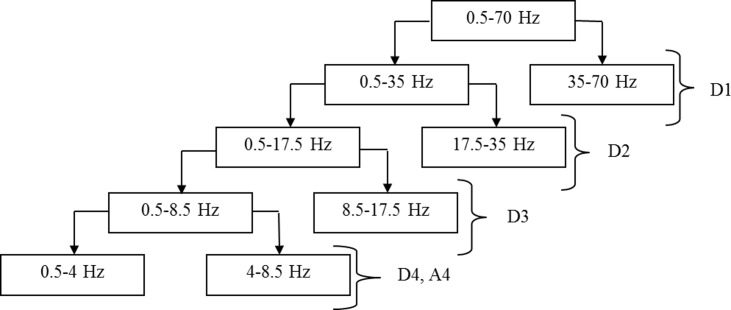
Multi-resolution decomposition of EEG signal (delta and theta bands) into detail and approximate coefficients.

Fig 4 shows multi-resolution decomposition of recorded EEG signals into corresponding detail and approximate wavelet coefficients based on Daubechies (db4) wavelet window function. The selection of this particular window function was motivated by the highest classification accuracy achieved when compared with other wavelet window functions. Moreover, the db4 provides near-optimal time-frequency location properties [68].

In this study, the WT analysis was performed in Matlab (version 7) software with ‘*wavedec*’ function. Further, the WT analysis involved the convolution of EEG signals with different dilations and translations of a wavelet basis function, e.g., the Daubechies (db4) wavelet. The dilations have resulted into different scales of EEG signal and the translations provided the convolution results which were function of time, and resulted into detailed and approximate wavelet coefficients, accordingly.

As described in Table 3, the EEG signals were recorded involving frequencies between 0.5 to 70 Hz. Therefore, five levels of wavelet decomposition were sufficient to extract the desired EEG bands. The wavelet coefficients extracted during each level of decomposition corresponded to individual EEG frequency bands such as the delta, theta, alpha, beta, and gamma (Table 3). In this study, wavelet coefficients from delta (A4) and theta (D4) bands were found most efficient (higher accuracies) than alpha, beta, and gamma bands while classifying treatment R and NR. Hence, the wavelet coefficients corresponding to alpha, beta, and gamma bands were discarded and were not considered while building the classifiers.

**Table 3:** Wavelet Coefficients in the delta and theta frequency bands.

Wavelet coefficients	EEG Frequency Bands	Frequency Range
**D1**	Gamma	35–70 Hz
**D2**	Beta	17.5–35 Hz
**D3**	Alpha + Low Beta	8.5 to 17.5 Hz
**D4**	Theta	4 to 8.5 Hz
**A4**	Delta	0.5 to 4 Hz

After performing the WT analysis, the extracted features were saved in the EEG data matrix. The columns of EEG data matrix corresponded to the EEG features such as the wavelet coefficients per channel (2825) × number of channels (19) = 53,675. Each EEG channel corresponded to 2825 wavelet coefficients representing delta and theta frequency bands (D4, A4). The rows of the data matrix (data points = 68) corresponded to the MDD patients data during EC and EO conditions. Finally, the resulting EEG data matrix dimension, i.e., the number of rows (data points = 68) were significantly less than the number of columns (number of observations per data point = 53,675). Table 4 provides the Matlab codes for the WT analysis.

**Table 4:** The Matlab code to compute the wavelet coefficients for delta and theta bands.

% The example code for decomposing a single EEG channel. A variable ‘Data’ has been assigned the de-artifacted EEG data with 1 channel.
1. Data = EEG_signal(1,:);
%Decomposition Levels
2. N = 5;
%Wavelet window function
3. wname = ‘db4’;
%Wavelet decomposition of the Data with 5 levels of decomposition using db4 window
4. [C,L] = WAVEDEC(Data, N, wname);
%Wavelet coefficients ‘A4’
5. Delta_band_coeff = C(1:L(1));
% Wavelet coefficients ‘D4’
6. Theta_band_coeff = C(L(1):L(1)+L(2));

#### Feature extraction: STFT analysis and EMD

In this study, the short-time Fourier transform (STFT) was computed by convolving a short-time squared window function with the EEG signal [69]. The Fourier transform of the windowed EEG signal was computed while traversing the whole EEG signal. The Fourier transform was computed based on the parameter values such as STFT window (hamming), Window length (2 sec), hop size (0.5 sec), number of fft points (4096 points, or 16 sec), sample frequency (256 samples/sec) and a 50% overlap between the squared window functions. The length of window function was selected such as to maintain the stationary nature of the EEG signal. In this study, a 2 second EEG segment was considered as stationery [70]. In this study, these parameter values were found optimal that provided best performance for STFT.

The empirical mode decomposition (EMD) involved decomposing the EEG signal into its subcomponents known as intrinsic mode functions (IMFs) [71]. EEG signal was decomposed into multiple IMFs using the parameters: Resolution (40 dB), residual energy (40 dB), gradient step size (1) [72]. An IMF can be computed by the following procedure: the peaks and troughs of the EEG signal were determined while inspecting its maxima and minima respectively. Based on these maxima and minima, the upper and lower envelops were constructed by cubic spline interpolation. Further, the mean of the upper and lower envelops was computed and subtracted from the EEG signal to obtain the probable IMF. An IMF should fulfill two conditions: 1) the number of extrema’s and zero-crossings must be equal or different not more than 1 and, 2) the mean of all IMFs must be zero or near to zero. In case an IMF was identified, it was subtracted from the EEG signal. The process of computing IMFs was repeated until each subsequent IMF was different from the previous one and fulfills the mean square error stopping criterion.

#### Feature extraction: Coherence and P300 components

In this study, the coherence was computed pair-wise between two different EEG electrodes and can be expressed by the following mathematical formula. According to the formula, the magnitude squared of the cross spectrum of two EEG sensors was computing and divided by a product of the power spectral densities (PSD) (PSD using Welch averaged periodogram method) of each of the signals as described in Eq (1):

$$C_{xy}(f)=\frac{{\left|S_{xy}\right|}^{2}(f)}{S_{x}(f)S_{y}(f)}$$ (1)

where *f* is the frequency, *S*_*x*_ is the PSD of *x*, *S*_*y*_ is the PSD of *y*, and *S*_*xy*_ is the cross-spectral density of the two EEG sensors of interest. The coherence was computed for each channel pair involving frontal (Fp1, Fp2, F3, F4, F7, F8, Fpz), temporal (T3, T4, T5, T6), parietal (P3, P4, P7, P8), occipital (O1, O2), and central (C3, C4). The coherence was computed for all possible pair combinations of EEG sensors over the scalp. In addition, the following parameter values were utilized such as 2 sec windows, 2 Hz-30 Hz band with 1 Hz resolution. Moreover, we have used the same feature selection and classification methods as used during the WT analysis.

In the event-related potential (ERP) data, the P300 peak was expected to appear between 300 to 700 milli-seconds after stimulus onset. In this study, the P300 amplitudes and latencies were computed by averaging the ERP data that corresponded to multiple target shapes or events of interest. Further, the data were grand averaged across all participants of one group in order to compare the P300 between the MDD patients and healthy controls. In addition, the computed values of P300 were utilized as input for the classification models.

#### Standardization

The EEG data matrix might not be centered and also unequally distributed. Therefore, in order to eliminate the possible outliers, and to improve classification performance, the data standardization based on z-scores was performed in Matlab (version 7) function ‘*zscore*’. For this purpose, the EEG data of second group (30 healthy control subjects) were used. The means *μ*_*l*_ and standard deviations *σ*_*l*_, *l = 1*,*…*, *N*_*c*_ for each feature were calculated over the healthy subject sample. Then for MDD patients, the corresponding *l-th* feature value *x*_*l*_ is replaced with its normalized z-score value $z_{l}=\frac{x_{l}-\mu_{l}}{\sigma_{l}}$ before being fed to the feature selection and classifier processes.

#### Feature selection

Most of the features extracted during feature extraction might be either redundant or irrelevant. Therefore, the feature selection is desirable to reduce dimensionality of the feature space, from *N*_*c*_ to a lower dimension, i.e., *N*_*r*_. For high dimensional data sets, feature selection remains as a challenging research topic and carries critical importance during data analysis involving a typical ML methodology. The high dimensional data/feature matrices have been commonly found during practical studies such as the research areas of Genetics and Chemo-metrics, where a large number of genes or compounds may be encountered typically within thousands to few millions. From the classification point of view, this high dimensionality may easily over-fit or under-fit a classification model. Hence, the high dimensionality causes a considerable deterioration of classifier performances. In addition, the larger the number of features used to describe the patterns in a domain of interest, the larger is the number of examples needed to learn a classification function to a desired accuracy [73, 74]. In this study, to enhance the classification performance and to reduce the irrelevancy and redundancy of the features, a rank-based feature selection method was used to select the most significant features from the EEG data matrix. In order to compare the rank-based feature selection method with a standard method, the study has employed minimum redundancy and maximum relevance (mRMR) method [67]. Hence, the classification results incorporated both types of feature selection methods.

The rank-based feature selection method was performed according to receiver operating characteristics (ROC) criterion [66, 75]. The area covered by the ROC curve for each feature indicated its relevance with the class labels such as more area under the curve (AUC), the higher is the relevance of that feature with the class label. Hence, the AUC was computed and a corresponding weight value (z-value) was assigned to each feature. The z-value was directly proportional to the area between the empirical ROC curve and the random classifier slope and may vary from 0 and 0.5 indicating bad to good classification ability, accordingly. A high z-value (equal or near 0.5) corresponded to the ability of a feature to discriminate within classes. After computing z-values, the features were arranged in descending order of the z-values such as the top-ranked features were listed at the top of the list. In order to eliminate the relevance among the top-ranked features, their correlations with each other were computed and the features with high correlation values were discarded because they might be redundant during classification. Hence, the discriminating features were obtained with the ROC-based feature selection based on the entire dataset (*N = 34*).

Table 5 provides pseudo code for the rank-based feature selection method to compute the AUC for an individual feature. Let x be a vector that represents a feature and the vector y represents the target labels (-1, +1). In this study, both x and y have same dimensions.

**Table 5:** Pseudo code for feature ranking method.

patterns = [x y];
patterns = sortrows(patterns,-1);
y = patterns(:,2);
p = cumsum(y = = 1);
tp = p/sum(y = = 1);
n = cumsum(y = = -1);
fp = n/sum(y = = -1);
n = length(tp);
Y = (tp(2:n)+tp(1:n-1))/2;
X = fp(2:n)—fp(1:n-1);
auc = sum(Y.*X)-0.5;

The sample data provided in Table 6 further explains the pseudo code for the rank-based feature selection method. Table 6 lists the sample data for 10 examples as shown in the first column. In addition, the columns ‘i’ and ‘j’ represent 2 different features, accordingly. The last column shows the corresponding class labels.

**Table 6:** Sample data.

Sample ID	…	i	j	…	Label
1		-0.2	+0.5		(-)
2		-1.4	-1.4		(-)
3		+0.8	-0.9		(-)
4		-0.8	+0.2		(+)
5	…	+0.1	-2.5	…	(+)
6		+0.5	+1.4		(-)
7		+1.6	-0.3		(+)
8		-2.1	-1.2		(-)
9		-0.3	+2.2		(+)
10		+3.4	-1.7		(-)

Tables 7, 8 and 9 lists the intermediate values of different variables during the computation of the AUC for the feature ‘i’ (as listed in Table 6). The computations follow the pseudo code provided in Table 5. As shown in Table 8, the first step is to sort the feature values in a descending order (1^st^ column) and the corresponding labels are also adjusted (2^nd^ column), accordingly. Further, the values of intermediate variables (i.e., p, n, tp, and fp) are computed and listed in the respective column.

**Table 7:** Intermediate variables values.

Feature values (sorted in descending order)	labels	p	n	tp	fp
3.4	(-)	0	1	0	0.1667
1.6	(+)	1	1	0.25	0.1667
0.8	(-)	1	2	0.25	0.333
0.5	(-)	1	3	0.25	0.5
0.1	(+)	2	3	0.5	0.5
-0.2	(-)	2	4	0.5	0.6667
-0.3	(+)	3	4	0.75	0.6667
-0.8	(+)	4	4	1	0.6667
-1.4	(-)	4	5	1	0.8333
-2.1	(-)	4	6	1	1

**Table 8:** Computation of Y = (tp(2:n)+tp(1:n-1))/2.

tp(2:n)	0.25	0.25	0.25	0.5	0.5	0.75	1	1	1
tp(1:n-1)	0	0.25	0.25	0.25	0.5	0.5	0.75	1	1
(tp(2:n)+tp(1:n-1))/2	0.25	0.5	0.5	0.75	1	1.25	1.75	2	2
Y	0.125	0.25	0.25	0.375	0.5	0.625	0.875	1	1

**Table 9:** Computation of X = (fp(2:n)-fp(1:n-1)).

fp(2:n)	0.1667	0.333	0.5	0.5	0.6667	0.6667	0.6667	0.8333	1
fp(1:n-1)	0.1667	0.1667	0.333	0.5	0.5	0.6667	0.6667	0.667	0.8333
X	0	0.1667	0.1667	0	0.1667	0	0	0.1667	0.1667

Table 8 provides the computation of the intermediate variable Y according to the formula Y = (tp(2:n)+tp(1:n-1))/2. Table 8 shows computation of Y based on values in Table 8.

Similarly, Table 9 provides the computation of the intermediate variable X according to the formula ‘X = fp(2:n)—fp(1:n-1)’. Table 9 shows the computed X based on the values obtained in Table 8.

Finally, the AUC is computed base on the formula ‘auc = sum(Y.*X)-0.5’. Table 10 shows the detailed values of intermediate variables Y and X and the AUC, respectively.

**Table 10:** Computation of AUC = sum(Y.*X)-0.5.

AUC = sum(Y.*X)-0.5;
AUC = (0.125×0 +0. 25×0.1667 + 0.25×0.1667 + 0.375×0 + 0. 5×0.1667 + 0.625×0 + 0.825×0 + 0.1667×1 + 0.1667×1)-0.5
**AUC = 0.5–0.5 = 0**

As shown in Table 10, the value obtained for AUC (z-value) is zero which means that the feature ‘i’ would not be a good option for further classification process and could be rejected. The process is repeated for all other features in the EEG data matrix.

Furthermore, a second technique of feature selection was employed, known as the minimum redundancy and maximum relevance (mRMR) [67]. According to mRMR, the most discriminant features were identified based on the measures such as maximum relevance and minimum redundancy. For example, the maximum relevant features were those that share maximum value of mutual information between the feature and the target labels. On the other hand, the features with minimum redundancy were identified based on the principle that if two features are highly dependent on each other, the respective class discriminative power would not change much if one of them is removed.

In order to find minimum number of features that would be sufficient to train the classifier model without over-fitting, an empirical process was adopted. According to the process, the minimum number of features were determined based on iteratively observing performance of the classification models for each feature subsets selected from top 1, 2, 3, 4, 5, 10, 15, 20, 25, 30, 35, and 50 features. In order to generate a sufficient statistical distribution of classifier performance metrics such as the accuracy, sensitivity and specificity for each subgroup, 100 times simulations were performed and box-plots were plotted.

In this study, the EEG features for EC and EO were combined by concatenating the individual feature columns-wise: 15 best features of WT + 15 of best STFT + 15 of best EMD features to make 45 features in total and then feed them to classifier.

#### Classification

In this study, a multivariate relationship between the EEG-based features and the clinical outcomes, i.e., R and NR was modeled based on logistic regression (LR) model [76]. The reduced set of EEG features was considered as independent variables and the corresponding treatment outcomes (R or NR) were the dependent variables. Logistic function provides the mathematical base on which the logistic model is based and is given by Eq (2):

$$F(z)=E(Y/x)=\frac{1}{1+e^{-z}}$$ (2)

where *Y* is the class labels and assigned a value of either ‘R’ or ‘NR’, and *x* represent a combination of the EEG features after feature selection, i.e., the coefficients achieved by WT technique and the features extracted from EMD and STFT analysis. To obtain the LR model from the logistic function, we used Eq (3):

$$z=\alpha +\beta_{1}X_{1}+\beta_{2}X_{2}+\ldots +\beta_{k}X_{k}$$ (3)

where z is a linear combination of α plus β_1_ multiplied with X_1_, plus β_2_ multiplied with X_2,_ and plus β_k_ multiplied with X_k_, where the X_k_ are the independent variables and α, and β_i_ are constant terms representing unknown parameters. Furthermore, by replacing the value of z from Eq (3) to Eq (2), the following Eq (4) represents the logistic function:

$$F(z)=E(Y/x)=\frac{1}{1+e^{-(\alpha +\sum \beta_{i}X_{i})}}$$ (4)

The likelihood of a person to be a non-responder or a responder was estimated and that resulted into a likelihood value *F(z)*, where *0 ≤ F(z) ≤ 1*, which was an indication of subject’s association with either R or NR category. If *F(z)* was greater than the *threshold = 0*.*5*, the subject was declared as R (responder), and otherwise as a NR (non-responder). In summary, the LR classifier generated probability values to categorize the MDD patients as either R or NR to the treatment.

#### Validation

The validation of classification results is provided by 100 iteration of 10-fold cross validation (10-CV) including a permutation test method [77]. Permutation tests were suggested in the evaluation of classification performance [78, 79]. After classifier design, a fair evaluation requires assessment of its performance over a range of selected features, data points (study participants) and classifier design that corresponds to a large number of subjects. To address this consideration, we evaluated classification performance based on 10-CV. The data points (study participants) were segmented such that during each round, nine of the segments were utilized as training subset and the remaining 1 as test subset.

For each feature subset, a 100 times run of the simulations were performed involving 10-CV to achieve box-plot representations of the accuracies, sensitivities and specificities. Since the individual iteration resulted into 100 different values of performance metrics (the accuracy, the sensitivity and the specificity), the final confusion matrix was computed by averaging. The performance metrics computed from the confusion matrix were presented by Eqs (5–8). The sensitivity of a classification model corresponds to the percentage of true cases (TP) which are correctly classified as cases defined by Eq (5). The specificity of a classification model refers to the percentage of true non-cases (TN) which are correctly classified as non-cases as described by Eq (6). The accuracy of a classification model illustrates the percentage of correctly classified cases and non-cases among all the example points as depicted in Eq (7). F-Measure, as described in Eq (8), could be interpreted as a weighted harmonic average of precision and recall values [80]. The precision was defined as the probability that a randomly selected patient analyzed to be MDD was really MDD patient. The recall was defined as the probability that a randomly selected MDD patient was correctly identified as a MDD patient. The F-Measure indicated that both the precision and recall were reasonably high.

$$Sensitivity=\frac{TP}{TP+FN}$$ (5)

$$Specificity=\frac{TN}{TN+FP}$$ (6)

$$Accuracy=\frac{TP+TN}{TP+TN+FP+FN}$$ (7)

$$F-Measure=\frac{2\times TP}{(2\times TP)+FP+FN}$$ (8)

### Construction of 2D maps of scalp topographies

In this study, the 2D topographic maps were constructed based on assigning values, i.e., either 0 or 1 and a corresponding color to each of 19 scalp locations involving the Wilcoxon rank-sum test [81, 82]. The Wilcoxon rank-sum test assigned values such as either ‘1’ or ‘0’ to each location showing the statistical differences between the two groups, i.e., the R and NR. Since the construction of topographic maps required values for 19 scalp locations, and 11 of them were listed in Table 3 (Fp2, F3, F4, F7, F8, Fz, C3, C4, P4, T3, T4). Therefore, the remaining locations such as Fp1, O1, O2, P3, T6, T8, Cz, Pz were determined from the 100 top-ranked features. The wavelet features corresponding to delta and theta bands were used to compute values for each scalp location.

According to the Wilcoxon rank-sum test, the null hypothesis (*H = 0)* stated that the medians of the two groups (R Vs. NR) were equal, and assigned a ‘0’ value and blue color for the location. On the other hand, the alternate hypothesis (*H = 1)* indicated a significant difference (not equal) at the 5% level and correspondingly assigned ‘1’ value and a red color for the location. The space between the two sensors was assigned a color by method of interpolating values of the two nearest sensor locations. As a result, the topographical maps for the 19 channels were constructed. The Wilcoxon rank-sum test was performed using a Matlab (version 7) function ‘*ranksum*’. In this study, the construction of 2D maps was performed in EEGlab [83], involving a Matlab function ‘*topoplot*’.

In this study, the particular selection of the Wilcoxon rank-sum test was based on the test of normality of the selected wavelet coefficients involving the kolmogrov-smirnov test [84, 85]. According to the kolmogrov-smirnov test, the analysis of variance (ANOVA) test was not feasible; therefore, an equivalent non-parametric test was chosen. In this study, to examine agreement between the distribution of the reduced set of EEG features and a normal distribution, the Kolmogrov-Smirvon (KS) test was performed. The KS test returns a test decision for the null hypothesis that the data in vector *x* (EEG features) comes from a standard normal distribution, against the alternative that it does not come from such a distribution. The test resulted into a value ‘1’ if the the null hypothesis was rejected at the 5% significance level, or into a value ‘0’ otherwise. The KS test was implemented using a Matlab (version 7) function ‘*kstest*’.

The gender stratification was recommended useful to elucidate the brain regions that could not be highlighted otherwise [86]. In order to realize the importance of gender stratification, the topographical maps were constructed without gender stratification as well.

### 2D scatter plotting with KPCA

In this study, the feature selection resulted into a reduced subset of the most discriminant features involving both the R and NR groups. To visualize a 2D representation of the data, a reduced set of EEG data matrix was computed involving the kernelized principal component analysis (KPCA) method [87]. The KPCA method was implemented involving a Matlab (version 7) function ‘*princomp*’. The method transformed the EEG data matrix into its principal components representing the variance of data. Specifically, the first two principal components depicted more than 80% variance of the EEG data and were plotted on the x-axis and y-axis, respectively. The resulting scatter plot diagram represented the distribution of R and NR classes in 2D space. The shape of the scatter plot helped in visualizing clustering behavior of the feature set and aids in identifying the outliers. In the scatter plot, each point corresponds to the epochs (2×34 = 68) involving all the MDD patients for EC and EO EEG data.

## Results

### Significance of wavelet coefficients and clustering behavior

To observe an overall behavior based on both MDD male and female patients, Table 11 lists top-ranked 15 wavelet features in delta and theta bands sorted in descending order according to their individual computed z-values. The p-values implicated that the wavelet features showed statistically significant difference between the R and NR groups. To summarize, among the top-rated 15 significant wavelet coefficients, nine of them were computed from the frontal lobe while three were found associated with temporal. The parietal and central areas had one and two coefficients, respectively. Based on the number of coefficients, it may be concluded that the frontal and temporal brain regions have shown most significant features in order to discriminate the two groups which is in accordance to the study conducted by [88].

**Table 11:** A list of discriminating features (Frontal = 9, Temporal = 3, Parietal = 1 and Central = 2).

EEG Electrode Names	Frequency band	Absolute z-values	p-value
**Fp2**	Delta	0.3024	0.016
**C3**	Theta	0.2886	0.022
**F7**	Delta	0.2794	0.013
**F3**	Delta	0.2794	0.022
**F7**	Theta	0.2739	0.016
**T4**	Theta	0.2711	0.022
**F8**	Theta	0.2711	0.008
**T4**	Delta	0.2711	0.010
**F3**	Theta	0.2665	0.002
**Fz**	Delta	0.2656	0.045
**F4**	Delta	0.2638	0.0021
**C4**	Delta	0.2601	0.015
**F8**	Theta	0.2574	0.021
**T4**	Delta	0.2555	0.030
**P3**	Theta	0.2555	0.001

**Fig 5:**
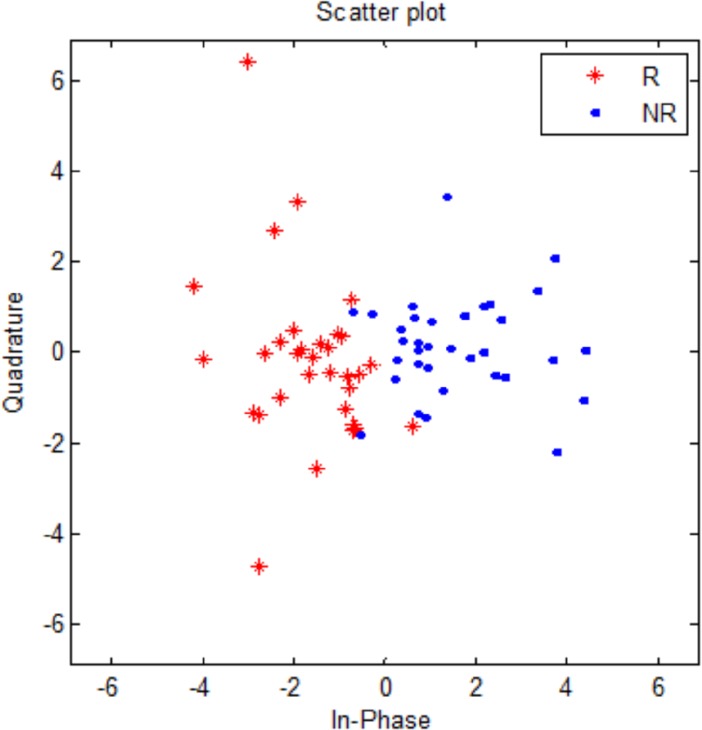
Scatter plot representation of first two PCs representing clustering behavior of the treatment responders (R) and non-responders (NR) achieved by kernelized principal component analysis (KPCA).

Fig 5 shows distribution of responders and non-responders on a 2D plane for the top-ranked 15 wavelet features. As shown in the figure, the shapes of the two clusters provide a bird’s eye view of the data and indicated that there were no outliers in this reduced set of EEG data.

### Topographic maps

**Fig 6:**
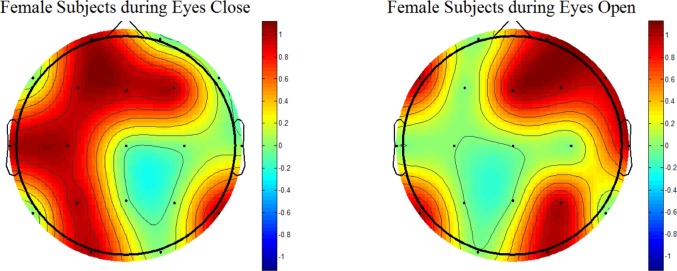
Wavelet coefficient based statistical differences between responders and nonresponders (Females Patients Only). According to the topo plots the left temporal areas showed significant differences during EC and EO conditions. The statistical difference of activation, between R and NR, was found in the frontal and central regions also.

**Fig 7:**
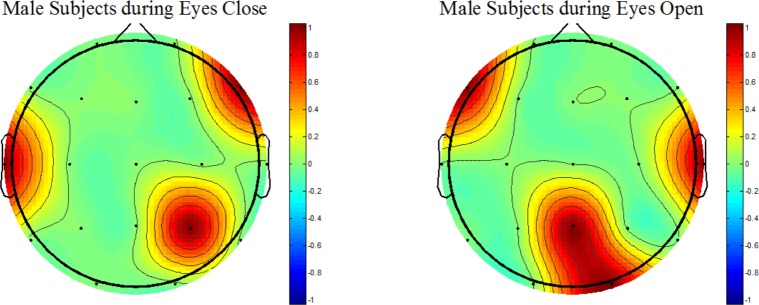
Wavelet coefficient based statistical differences between responders and nonresponders (Both male and female Patients Only). During EC, left and right temporal areas as well as the left frontal have shown significant differences. In addition, during EO condition the frontal, right temporal and central and parietal areas were also exhibited significant differences.

Figs 6 and 7 show topographic maps constructed for MDD female and male patients, respectively. Fig 6 (left-side) shows the MDD female patients during EC. Brain regions such as the frontal, left and right temporal have shown significant differences between the R and NR. In addition, some other areas such as left central, parietal and occipital have also exhibited significant differences. In Fig 6, during EO (right-side), the MDD female patients have exhibited differences between female R and NR in the right frontal and temporal areas. In addition, a right sided occipital and parietal have shown significant difference. In short, during both EC and EO conditions, frontal and temporal areas were commonly observed as significantly different between the two groups.

In Fig 7, the male participants during EC (left-side) had exhibited statistically significant differences in the right frontal, left temporal and right parietal regions. During EO (right-side), in addition to the frontal and temporal areas, the central and parietal regions have shown the statistical differences. Similar to the female patients, it was observed that the frontal and temporal regions were common between the EC and EO conditions.

**Fig 8:**
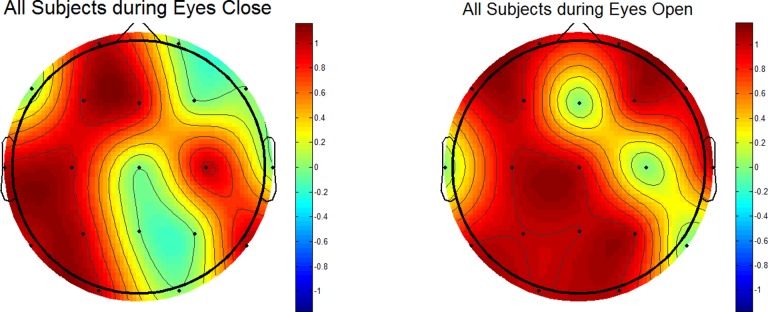
Wavelet coefficient based statistical differences between responders and non-responders (All MDD patients). The figure shows significance of gender stratification for topographical analysis.

The Fig 8 was plotted without gender stratification including the EC (left) and EO (right) conditions. During EC (left-side), statistical differences were observed in the right frontal, left and temporal and right parietal regions. During EO (right-side), except some small areas, the whole brain region has shown the statistical difference.

### Classification of MDD patients based on significant wavelet coefficients

Table 12 provides comparison of the proposed ML method with state-of-the-art methods as mentioned in the ‘Introduction’ section including power of EEG bands, i.e., alpha and theta power, alpha asymmetry, ATR index, EEG theta cordance, coherence and P300 amplitude and latencies. The number of features reported here has shown maximum classification accuracies with the given feature set. According to the Classification results, the proposed ML method out-performed the existing state-of-the-art methods. The second best accuracy was achieved by the P300 amplitude and latencies, i.e., 74.16%. However, the associated specificities are very low.

**Table 12:** Comparison of classification (R Vs NR) methods among the proposed ML method and the methods presented in the related literature.

Derived EEG Measure	Accuracy	Sensitivity	Specificity
**ATR Index [18]**	61.68% (±9.1)	70% (±15.3)	54% (±17.2)
**EEG Theta Cordance [19]**	70.7% (±6.5)	75.7% (±9.1)	65.7% (±8.3)
**Coherence, PSD, PSD ratio [31]**	72.08% (±7.6)	80% (±13.5)	65% (±12.3)
**P300 (amplitude and latencies) [89]**	74.16% (±13.1)	70% (±15.6)	75% (±7.7)
**PSD, PSD ratios [31]**	54.5% (±8.0)	55% (±14.7)	50% (±15.6)
**Proposed ML Method**	87.5% (±7.1)	95% (±4.3)	80% (±8.8)

**Fig 9:**
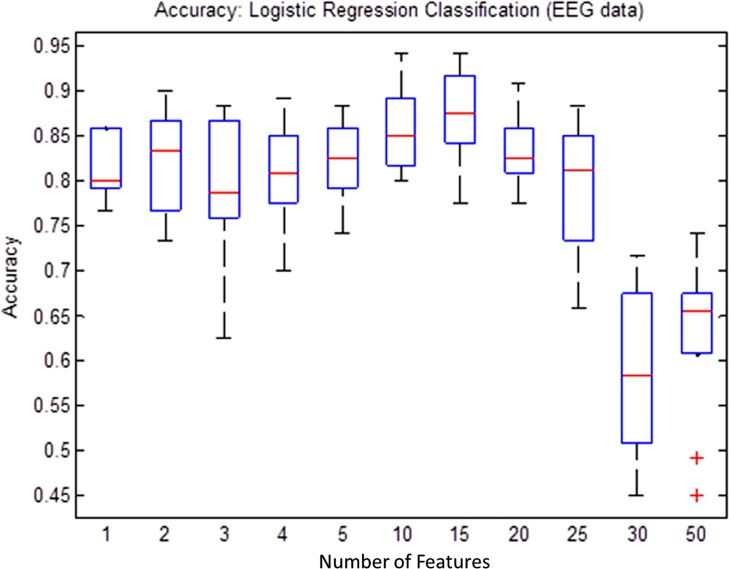
Classification accuracies (Logistic Regression (LR)) as a function of number of features. Over-fitting can be observed by a decrease in accuracy (more than 15 features) with an increase in the number of features.

Fig 9 shows classifier performances as a function of the total number of features in a subset. The LR classifier has exhibited an over-fitting phenomenon because the accuracy of the classifier decreases with an increase in the number of features. According to the figure, the top 15 features exhibited highest efficiencies. Increasing the features more than 15 resulted into a decrease in the classification performance.

Table 13 provides comparison of the three time-frequency decomposition techniques including WT analysis, STFT and EMD while classifying the treatment R and NR. According to the results, the EEG features computed with WT analysis have shown highest classification efficiencies (accuracy = 87.5%) among other EEG features. In addition, the rank-based feature selection showed better results than the mRMR. On the other hand, the STFT and EMD based EEG feature extraction have shown lower performance than WT analysis. An integration of the features including WT analysis, EMD, and STFT as a single features space matrix has shown accuracy = 91.6%.

**Table 13:** Classification (R vs. NR) for EEG Data including Delta and Theta Wavelet coefficients.

EEG Features	Feature Selection	Classification Performance			
		Accuracy	Sensitivity	Specificity	F-Measure
**Wavelets**	Rank Based	**87.5% (±7.1)**	**95% (±4.3)**	**80% (±8.8)**	**0.81 (±2.1)**
	mRMR	72.0% (**±**7.3)	70% (**±**9.9)	77.5% (**±**12.2)	0.70 (**±**2.8)
**STFT**	Rank Based	80% (**±**11.1)	75.0% (**±**10.5)	90% (**±**8.7)	0.76 (**±**4)
	mRMR	70.8% (**±**9.2)	60% (**±**14.8)	75% (**±**12.1)	0.64 (**±**4.9)
**EMD**	Rank Based	72.5% (**±**9.1)	77.5% (**±**17.7)	67.5% (**±**10.1)	0.73 (**±**5.2)
	mRMR	64.1% (**±**9.3)	60.0% (**±**9.2)	70.0% (**±**12.1)	0.52 (**±**1.3)
**Combination (Wavelets+STFT+EMD)**	Rank Based	**91.6% (±3.2)**	**90% (±9.4)**	**90% (±8.9)**	**0.86 (±1.7)**
	mRMR	76.2% (**±**8.6)	72.5% (**±**10.8)	77.5% (**±**11.7)	0.74 (**±**1.9)

**Fig 10:**
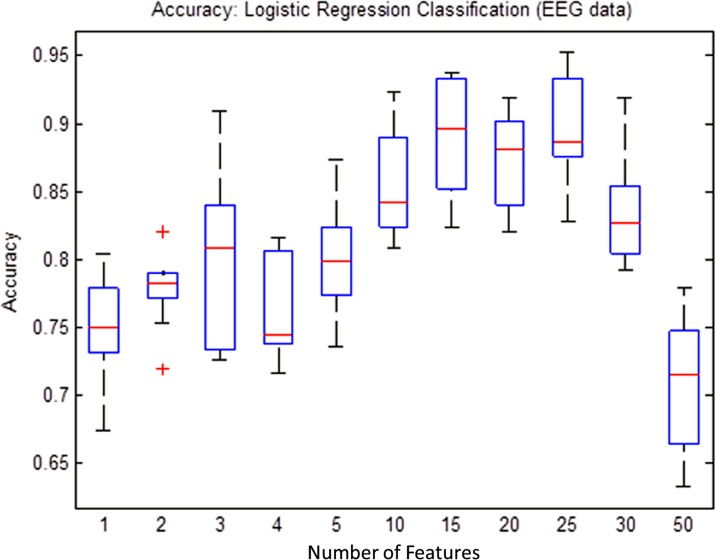
Classification accuracies (Logistic Regression (LR)) as a function of number of features. Over-fitting can be observed by a decrease in accuracy (more than 15 features) with an increase in the number of features.

Fig 10 shows the results for training and testing the LR classifier for each subset of features while classifying the MDD patients and healthy controls. As shown in the figure, each plot shows classifier performance as a function of the total number of features in a subset. The LR classifier has exhibited an over-fitting phenomenon that can be observed from the figures as the accuracy of the classifier decreases with an increase in the number of features. According to the diagram, the top 15 features have exhibited highest efficiencies. Increasing the features greater than 15 resulted in a decrease of classification performance.

Table 14 provides comparisons of the three time-frequency decomposition techniques including WT analysis, STFT and EMD while classifying the MDD patients and healthy controls. The EEG features computed with WT analysis have shown highest classification efficiencies (accuracy = 89.6%) among other EEG features. In addition, the rank-based feature selection showed better results than the mRMR. On the other hand, the STFT and EMD based EEG feature extraction have shown lower performance than WT analysis. The detailed results can be seen from the table. An integration of the features including WT analysis, EMD, and STFT as a single features space matrix has shown accuracy = 90.5%. Studies using the similar idea of using the ML methods have reported diagnosis accuracy such as [90, 91].

**Table 14:** Classification (MDD patients vs. healthy controls) for EEG features including Delta and Theta Wavelet coefficients.

EEG Features	Feature Selection	Classification Performance			
		Accuracy	Sensitivity	Specificity	F-Measure
**Wavelets**	Rank Based	**89.6% (±5.1)**	**81.7% (±11.3)**	**96.7% (±3.1)**	**0.77 (±2.9)**
	mRMR	65.2% (**±**4.2)	63.3% (**±**6.4)	68.7% (**±**9.5)	0.62 (**±**1.9)
**STFT**	Rank Based	82.2% (**±**8.8)	80% (**±**14.1)	82.9% (**±**9.2)	0.83 (**±**2.9)
	mRMR	62.5% (**±**5.5)	63.3% (**±**9.1)	60.8% (**±**6.5)	0.58 (**±**2.9)
**EMD**	Rank Based	71.7% (**±**7.4)	68.3% (**±**16.1)	77.1% (**±**14.1)	0.56 (**±**14.6)
	mRMR	56.1% (**±**9.1)	56.6% (**±**11.9)	51.6% (**±**9.2)	0.52 (**±**2.8)
**Combination (Wavelets+STFT+EMD)**	Rank Based	**90.5% (±8.3)**	**91.6% (±5.7)**	**88.7% (±7.5)**	**0.84 (±3.6)**
	mRMR	73.9% (**±**6.6)	75% (**±**7.8)	74.1% (**±**9.5)	0.70 (**±**1.9)

## Discussion and conclusion

In this paper, a ML method is proposed involving time-frequency decomposition of EEG data with WT analysis. A primary finding is that the pre-treatment EEG-based wavelet features involving delta and theta frequency bands can predict antidepressant’s treatment outcome for MDD patients treated with SSRIs. On the other hand, in psychiatric clinics, treating MDD is an iterative process with hit-and-trial sequential treatment strategy, until an effective antidepressant is found. In case of treatment failure, a two to four weeks’ time is wasted. This conventional clinical practice may be improved by incorporating EEG data because the scientific predictions based on electrophysiological recordings may help psychiatrists to evaluate the most appropriate antidepressant. Moreover, the successful predictions may effectively improve treatment process while reducing the useless treatment iterations.

In this study, utilizing EEG features as input data to the proposed ML method to perform classification of treatment respondents and non-respondents is based on the findings reported by [32]. However, the proposed ML method offers new methodology that provides high efficiency (accuracy, sensitivity and specificity) with less features, i.e., only 15 wavelet coefficients. The decomposition of EEG data at various scales has been considered as direct representations of the brain behavior at various scales with timing information [92, 93]. In comparison to the techniques proposed in literature, our method has shown highest efficiencies in discriminating R and NR. For example, recent studies based on ML concepts have shown 85% [31], and 87.9% [32], while our proposed method shows 87.5% accuracy. In addition, our proposed scheme is different in terms of extracted features, feature selection and classification models. We have employed 10-fold cross validation similar to the paper as employed by [32].

In this study, the brain areas such as frontal, temporal, parietal and occipital were identified as significantly different between the study groups. This finding is in accordance with other research studies related to MDD [94]. More specifically, the finding in visual cortex is interesting as some previous studies have reported functional abnormalities within the visual cortex in depression [95, 96]. Other studies based on structural observation such as MRI including MDD patients with abnormalities associated with frontal, temporal, parietal and occipital regions [94, 97–99]. However, the main contribution is that our data has replicated these findings with wavelet coefficients. In addition, our findings suggest that EEG could be used to assess treatment efficacy involving most relevant EEG features.

While constructing the topographical maps, the activation refers to the statistical differences between the brain areas of treatment responders and non-responders. The statistical differences are color-coded as red and blue corresponding to 1 (activation) and 0 (no-activation), respectively. In this study, the MDD patients are stratified as male and female MDD patients. It has been established that the gender stratification could help identifying the brain areas that could not be revealed otherwise [86]. In the literature, it is reported that gender differences affect pathological brains, including the subjects with subclinical depression and MDD [100]. In an old study, gender differences in the EEG activity during stimulus and non-stimulus conditions are also reported [101]. However, the proposed method has incorporated customized wavelet coefficients for this replication of previous findings. The results further motivate the use of topographical maps based on EEG data to localize brain regions that are different between the MDD responders and non-responders. The brain areas such as frontal, temporal, occipital, and parietal have shown significant associations with the disease pathology. This finding implicates that the topographical maps constructed with statistical quantities could be utilized to localize the disease pathology with a certain level of confidence. This finding would be of interest for the clinicians.

According to our topographic analysis, a gender difference is statistically significant between R and NR stratified into male and female MDD participants specifically at frontal and temporal brain regions. As shown in Figs 6, 7 and 8, while constructing the topographical maps, the significance of gender stratification is evident. Because only Figs 6 and 7 are able to show those brain regions which could not be manifested without gender stratification (shown in Fig 8). According to the literature, the gender differences in prevalence of depression is well-established which is found 2:1 in females as compared with male patients [102]. In addition, the gender difference is commonly found in terms of clinical features such as female patients report greater severity of illness and are more likely to receive the previous treatment for depression than male patients [103]. Moreover, greater functional impairment is noticed in women during marital adjustments whereas the men show more functional impairment during work-related issues. Gender differences in clinical symptoms may have implications in the treatment planning which may be gender-specific. In short, the chronicity of depression may affect female MDD patients more seriously than the male MDD patients. The analysis of topographical maps have shown similar brain regions that are in accordance with the literature [88]. However, our main contribution is that we have produced these topographical maps based on customized wavelet coefficients from pretreatment EEG data of patients recruited in this study.

The ATR [18] method predicts antidepressant’s treatment outcome with ~74% accuracy. However, the method suffers from the disadvantage that it can predict the treatment outcome based on the data acquired during week 0 (pretreatment) and 1 (one week after treatment start). Moreover, the EEG theta cordance and the ERP-based techniques, i.e., P300 and loudness dependence auditory evoked potential (LDAEP) resulted in low values of specificities [104, 105]. In contrary, the method we presented in this paper provides higher values of specificities after only a single pretreatment EEG data that favors its clinical utility.

While comparing power spectral density (PSD) and WT analysis, the PSD is computed while averaging the high resolution EEG data which would eliminate the temporal information. On the other hand, the WT analysis decomposes the EEG signal while preserving both time and frequency information. Therefore, in our study, the WT analysis is preferred over estimating PSD of the EEG signal. Moreover, in Table 13, the classification results for both the PSD and PSD rations have shown that the power computed with Welch’s averaged periodogram method are able to acquire 54.5% classification accuracy. In contrary, the WT analysis exhibits 87.5% accuracy. Hence, WT analysis performs better than the PSD estimation for the EEG data acquired in this study. Moreover, the quantification of connectivity among different brain regions is performed using the coherence measure. Regarding the effects of EEG data lengths on EEG analysis, we observed slight changes in classifier performances as a function of EEG data lengths. Hence, recommending the use of two minutes of EEG data that would perform better in terms of classification accuracy than one minute recording of EEG data.

In this study, wavelet coefficients extracted from delta and theta bands have shown higher efficiencies in discriminating the two study groups than the wavelet coefficients extracted from alpha and beta bands. In addition, the wavelet coefficients from frontal, temporal, and occipital regions are found significant. The neurobiology of MDD associated with EEG delta and theta band and with the frontal region can be explained: the theta current density, localized by LORETA to the rostral anterior cingulate cortex (rACC), has been associated with response to various antidepressants including, nortriptyline, citalopram, reboxetine, fluoxetine or venlafaxine during depression [28–30]. Pizzagalli has demonstrated biological mechanisms for this association [106]: According to Pizzagalli, the rACC has been considered as a main hub within the default network (DN) of the brain and involved in self-focused processing. Moreover, elevated resting state activity in rACC is associated with focusing on reflective thought or task independent introspection such as rumination, remembering and planning [107]. Rumination is a mechanism of responding to distress by repetitively focusing on the symptoms, causes and consequences of distress, and it is comprised of two components: reflective pondering and brooding. Cognitive problem solving is carried out through reflective pondering whereas the brooding is analytic self-focus, which is ultimately destructive because it worsens depressive symptoms. Based on these findings, Pizzagalli proposes that elevated rACC activity may lead to treatment response because of adaptive self-referential functions such as mindfulness and non-evaluative self-focus. Moreover, the rACC functional connectivity is observed in MRI study that demonstrated the discriminative power of rACC functional connectivity in depression [108].

There is a possibility that our proposed ML models are confounded with some outliers other than the relevant patterns extracted from the brain activities. We have ruled out this concern by 1) properly adopting artifact removal techniques, 2) standardizing preprocessed data based on z-scores, 3) plotting the low dimensional representation of our feature space: this helps in identifying outliers which may disturb the interpretations and conclusions, 4) during classifier’s testing and training, selecting random data points so that each data point in the feature space can be used, 5) in terms of classification, equally distributing both the R and NR classes within MDD male and female patients. Based on all these precautions, we may conclude that the results shown here are un-biased and true representation of the information from the recorded pretreatment EEG data.

The study is confounded with few limitations. During our MDD patient recruitment, it is difficult to recruit patients under a common treatment. As a result, the inclusion of patients is restricted to a single class of antidepressants i.e., SSRIs. Since the pharmaco-EEG profiles of different antidepressants are not clear yet, therefore, it is difficult to study medication-specific treatments effects. A potential confounding of head motion should be considered in caution, since both the neuronal and noise effects of head motion have been demonstrated to relate to the frontal and temporal regions, while head motion levels are always significantly different between different populations [109]. In this study, the results are based on small sample sizes, the generalizations of the results are necessary based on replicating our method into larger population. The study patients are required to be in washout for a period of two weeks before first EEG data recording session. However, the possibility of medication effects cannot be avoided completely. In future studies, inclusion of psychophysiological characteristics integrated with EEG may improve prediction performance.

In conclusion, despite the above mentioned limitations, the higher efficiencies shown in the results suggest that wavelet features from delta and theta bands might be a promising tool for prediction of therapeutic actions for SSRIs treatment. Specifically, the high specificities achieved by our method are of considerable interest for their clinical utilities. However, caution must be adopted while interpreting these results.
